# Complete Genome Sequence of *Bacillus subtilis* Strain ATCC 6051a, a Potential Host for High-Level Secretion of Industrial Enzymes

**DOI:** 10.1128/genomeA.00532-15

**Published:** 2015-05-21

**Authors:** Haeyoung Jeong, Young Mi Sim, Seung-Hwan Park, Soo-Keun Choi

**Affiliations:** a Super-Bacteria Research Center, Korea Research Institute of Bioscience and Biotechnology (KRIBB), Daejeon, Republic of Korea; b Biosystems and Bioengineering Program, University of Science and Technology (UST), Daejeon, Republic of Korea; c Korean Bioinformation Center (KOBIC), Daejeon, Republic of Korea

## Abstract

*Bacillus subtilis* ATCC 6051a (=KCTC 1028), which is less domesticated than strain 168, is widely used for the secretory expression of industrial enzymes. Herein, we present the complete genome sequence of the *Bacillus subtilis* strain ATCC 6051a.

## GENOME ANNOUNCEMENT

*Bacillus subtilis*, the most widely studied Gram-positive bacterium, has long been used for biotechnology applications. Some *Bacillus* species, including *B. subtilis*, are generally regarded as safe (GRAS) organisms, which makes them feasible microorganisms for direct human use. *B. subtilis* is also widely used for the secretory expression of many industrial enzymes and pharmaceutical proteins.

Although *B. subtilis* strain 168 is considered a model system for *B. subtilis* (1), it carries many mutations occurring through domestication, such as mutagenesis from irradiation and selection (2). The complete genome sequence of the wild-type strain ATCC 6051^T^ was recently determined by a German group (3). In this study, ATCC 6051a (=KCTC 1028, also known as P31K6), a strain that is widely used for the secretory production of industrial enzymes (4–7), was chosen for genome sequencing and analysis. Although the strain name implies the possible derivation of ATCC 6051a from ATCC 6051^T^, we could not delineate the origin of ATCC 6051a from the literature, with the exception of the source information from the ATCC website. Differences in antibiotic sensitivity against amoxicillin and nalidixic acid, and fatty acid methyl ester analysis profiles suggests its distinctive feature of ATCC 6051a (http://www.ec.gc.ca/ese-ees/E35B18C1-E940-4204-9500-DAF41BA56F79/DSAR_Micro-organisms_Bacillus_EN.pdf).

The genome sequencing of *B. subtilis* ATCC 6051a was carried out using an Illumina HiSeq 2000 platform. Cells were purchased from the Korean Collection for Type Cultures. Library construction (average insert length of ca. 400 bp) and 101-cycle paired-end sequencing were carried out by the National Instrumentation Center for Environmental Management from Seoul National University (Seoul, Korea). A total of 33,192,942 reads (ca. 3.35 Gb) were quality trimmed, filtered by length, and mapped against the genome sequences of *B. subtilis* strains 168 and ATCC 6051 using the CLC Genomics Workbench version 6.5 (CLC bio). The complete genome sequence of ATCC 6051a was obtained by revising the reference sequences according to the quality-based variants identified by the mapping procedure. Ambiguous bases and larger indels were confirmed through the Sanger sequencing of the PCR products. The final genome sequence was reconfirmed through a remapping analysis, which did not detect any variants (single nucleotide variants [SNVs], indels, or structural variations) or read breakpoints. The *de novo* assembly of unmapped reads did not suggest the presence of any ATCC 6051a-specific genomic regions. Genome annotation was carried out using the NCBI Prokaryotic Genome Automatic Annotation Pipeline (PGAAP) service. A complete list of variants among strains 168, ATCC 6051, and ATCC 6051a is available from http://wiki.genoglobe.kr/kribb/ATCC_6051a.

### Nucleotide sequence accession number.

This complete genome sequence was deposited at DDBJ/EMBL/GenBank under the accession no. CP011115.
